# Negative refraction in twisted hyperbolic metasurfaces

**DOI:** 10.1515/nanoph-2021-0561

**Published:** 2021-11-30

**Authors:** Yi Liu, Chunmei Ouyang, Quan Xu, Xiaoqiang Su, Jiajun Ma, Jing Zhao, Yanfeng Li, Zhen Tian, Jianqiang Gu, Liyuan Liu, Jiaguang Han, Weili Zhang

**Affiliations:** Center for Terahertz Waves and College of Precision Instruments and Optoelectronics Engineering, and Key Laboratory of Optoelectronic Information Technology (Ministry of Education), Tianjin University, Tianjin 300072, China; Institute of Solid State Physics, College of Physics and Electronic Science, Shanxi Province Key Laboratory of Microstructure Electromagnetic Functional Materials, Shanxi Datong University, Datong 037009, China; School of Electrical and Computer Engineering, Oklahoma State University, Stillwater 74078, OK, USA

**Keywords:** negative refraction, non-diffraction transmission, surface wave, topological transition, twisted hyperbolic metasurface

## Abstract

Hyperbolic metasurfaces with unique dispersion properties can manipulate light–matter interactions according to the demands. However, due to their inherent physical properties, topological transitions (flat bands) exist only in the orthogonal directions, which greatly limit their application. Here, we unveil rich dispersion engineering and topological transitions in hyperbolic metasurfaces. Based on the effective medium theory, the rotation matrix is introduced into the dispersion relation to explain the distorted energy band diagrams, iso-frequency contours and higher-order multi-dipoles of the novel twisted metasurfaces, thereby forming multi-directional topological transitions and surface plasmon polariton propagation. Furthermore, we develop an integrated model to realize new dual-channel negative refraction and nondiffraction negative refraction. The phenomena observed in the experiments match well with the simulations, which proves that the designed metasurfaces make new types of negative refraction possible and will help to overcome the diffraction limit. The hyperbolic metasurfaces presented here exhibit exceptional capabilities for designing microscopes with a super lens at the molecular level, concealment of military aircraft, invisibility cloaks and other photonic devices with higher transmission efficiency.

## 1 Introduction

Topological dispersion in the momentum space, widely existing in hyperbolic metamaterials [1], [2], [3], [4], [5], [6], linear-crossing metamaterials [7], [8], [9], epsilon-near-zero media [10], [11], [12], and magic-angle graphene superlattices [13], [14], [15], [16], [17], results in a number of interesting optical phenomena such as self-collimating transport [18], [19], [20], anomalous wave propagation [21], [22], [23], negative refraction and so on [24], [25], [26], [27], [28]. Due to their low propagation loss and simple manufacturing process, hyperbolic metasurfaces (HMSs) have attracted substantial interest in manipulating surface plasmon polariton (SPP) propagation [21, 29]. Besides, based on their 2D characteristics HMSs show exceptional capabilities in planar optoelectronic devices [30], [31], [32] and have great potential in super-resolution imaging [33], [34], [35], wearable devices [36], [37], [38], [39], invisible cloaks [40, 41], etc. Recently, topological transition metasurfaces have attracted enormous research interests [42], [43], [44]. One of the most fascinating features of HMSs is that they have a topological dispersion exhibiting a flat line at the transition point, and such a property enables HMS to support propagating high-*k* modes and possess enhanced photonic density of states (hence a large Purcell factor), and promises fascinating applications, such as large field enhancement, sensing, and sub-diffraction imaging [3, 4, 39]. However, in the case of flat bands in the first Brillouin zone, the SPP transmission is self-collimated and confined in the orthogonal directions due to the intrinsic physical nature. Can the tuning of transport directions of surface waves be realized in HMSs?

Adjustable iso-frequency contours (IFCs) are a straightforward and effective manner in the manipulation of the light–matter interactions. However, recent developments in metasurfaces have concentrated on the control of polarization, phase and amplitude [45], [46], [47], [48]. In particular, tuning the shape of hyperbolic dispersion in an active manner determines the frequency of the phase transition. For example, in the microwave regime, 2D transmission lines [8, 49, 50], multilayers [51, 52], and metallic nanowire structures [29, 53] can be used to obtain topological transition in IFCs by varying the loaded capacitance and inductance. In the infrared regime, active tuning of the IFCs can be achieved by changing the loaded voltage of graphene or graphene dielectric multilayer structures, but the interlayer voltage is difficult to adjust artificially, which leads to great inconvenience to experimental measurements [54, 55]. Unlike the active manipulation mentioned above, there are also many examples of passive control. Multilayer fishnets can be tuned by changing the thickness of different layers or choosing different wavelengths in the visible regime [56, 57], and the research scope also has been extended to the terahertz and microwave regimes, which depends on the adjustment of IFCs by changing the permittivity, permeability, shape, or structural parameters of the metal–dielectric–metal structures and the metal–dielectric structures [22, 42, 58, 59].

In this article, an ultrathin integrated metasurface with hyperbolic dispersion is proposed and experimentally demonstrated. We take the unique electromagnetic characteristics such as energy bands and IFCs as starting points. From the dispersion properties, it can be found that topological transition frequencies only exist in the orthogonal directions. Here, an efficient method is proposed to control the multi-directional topological transition. When the proposed hyperbolic cells are rotated counterclockwise, the metasurface exhibits a higher-order coupling mode, thereby breaking the band overlap, and a new series of self-collimation frequencies emerge. At the same time, the corresponding energy bands and IFCs have also been rotated counterclockwise, so the metasurface promises a new platform to mimic diverse surface wave propagation processes. Finally, we have designed an integrated metasurface prototype to manipulate dual-channel negative refraction and nondiffraction negative refraction phenomena, whose unique transmission properties will benefit the hyperlenses, stealth materials and wearable devices.

## 2 Simulation and discussion

### 2.1 Structural design

**Figure 1:**
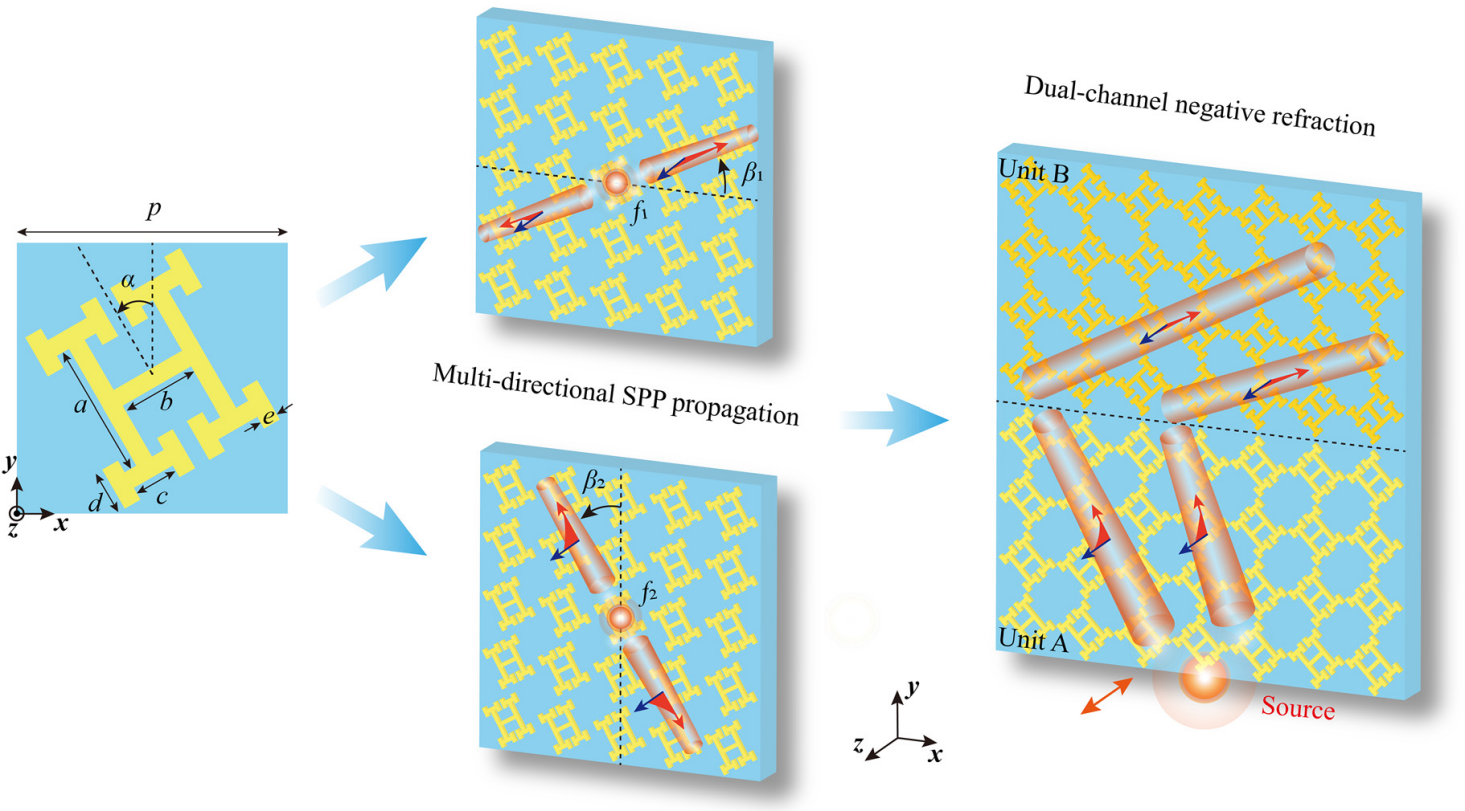
Schematic diagram of the proposed twisted HMSs consisting of a dielectric/Cu bilayer, where *p* = 9 mm, *a* = 4.5 mm, *b* = 2.6 mm, *c* = *d* = 1.6 mm, and *e* = 0.56 mm, and the counterclockwise rotation angle of the unit cell is *α*.

The sketch of a single unit of the designed metasurfaces is schematically shown in the left panel of Figure 1, where the parameters are: *p* = 9 mm, *a* = 4.5 mm, *b* = 2.6 mm, *c* = *d* = 1.6 mm, and *e* = 0.56 mm. To fulfill the requirement of rotation in the following steps, the parameters must meet the condition: ${\left(b+c+3\times e\right)}^{2}+{\left(a+e+d\right)}^{2}<p^{2}$ . In the middle panel, the copper patterns are periodically arranged on a lossless substrate (F_4_BM300) with a permittivity of 3.0, and the thicknesses of the dielectric layer and metal patterns are 1 and 0.035 mm, respectively. Surface waves at the topological transition frequencies along orthogonal directions of the first Brillouin zone are rotated along the horizontal and vertical directions when the unit cells rotate by the angle *α* (0 ≤ *α* ≤ 45°) counterclockwise, achieving multi-directional SPP propagation. Finally, as depicted in the right panel of Figure 1, an integrated model composed of arrays of units A (*α*_1_ = 45°) and units B (*α*_2_ = 135°) is established to realize dual-channel negative refraction and nondiffraction negative refraction when the SPPs reach the interface between the two different arrays of units and then propagate along two paths perpendicular to the original directions.

### 2.2 Basic physical properties

Based on the effective medium theory (EMT), for the electric HMSs, the relative permeability $\hat{\mu }$ is reduced to a unit tensor, and the relative permittivity tensor is given by [60, 61]:

(1) $$\hat{\epsilon }=\left(\begin{array}{ll} \epsilon_{xx} & 0\\ 0 & \epsilon_{yy} \end{array}\right)\text{.}$$

The subscripts *xx* and *yy* denote the components parallel to the *x* and *y* axes, respectively. The dispersion relation of the HMSs can be determined by substituting Eq. (1) into the Maxwell’s equations. In particular, for the TM mode, the dispersion relation can be written as:

(2) $$\frac{k_{x}^{2}}{\epsilon_{yy}}+\frac{k_{y}^{2}}{\epsilon_{xx}}=k_{0}^{2}\text{,}$$

where *k*_0_ is the wave-vector in free space, and *k*_x_, *k*_y_ are the *x* and *y* components of the wave-vector, respectively. Then, the rotation matrix $\hat{T}\left(\alpha \right)$ is adopted to the designed model to better understand the dispersion when the unit cell rotates counterclockwise at an angle *α* around the *z* axis:

(3) $$\hat{T}\left(\alpha \right)=\left(\begin{array}{ll} \cos\left(\alpha \right) & -\text{sin}\left(\alpha \right)\\ \text{sin}\left(\alpha \right) & \cos\left(\alpha \right) \end{array}\right)\text{.}$$

The relative permittivity tensor in Eq. (1) should be rewritten as:

(4) $${\hat{\epsilon }}_{T}=\hat{T}{\left(\alpha \right)}^{-1}\hat{\epsilon }\hat{T}\left(\alpha \right)\text{.}$$

In this case, the dispersion relation can be derived as:

(5) $$Ak_{x}^{2}+Bk_{y}^{2}+Ck_{x}k_{y}=\epsilon_{xx}\epsilon_{yy}k_{0}^{2}\text{,}$$

where $A=\cos^{2}\left(\alpha \right)\epsilon_{xx}+{\text{sin}}^{2}\left(\alpha \right)\epsilon_{yy}$ , $B={\text{sin}}^{2}\left(\alpha \right)\epsilon_{xx}+\cos^{2}\left(\alpha \right)\epsilon_{yy}$ , and $C=\text{sin}\left(2\alpha \right)\left(\epsilon_{yy}-\epsilon_{xx}\right)$ . When *α* = 90°, the dispersion relation Eq. (5) changes to:

(6) $$\frac{k_{x}^{2}}{\epsilon_{xx}}+\frac{k_{y}^{2}}{\epsilon_{yy}}=k_{0}^{2}\text{.}$$

We can clearly see that when the structural unit rotates 90°, the IFC rotates 90° accordingly. However, there are nondiagonal terms in the permittivity tensor when *α* = 0–90°:

(7) $${\hat{\epsilon }}_{T}=\left(\begin{array}{ll} \cos^{2}\left(\alpha \right)\epsilon_{xx}+{\text{sin}}^{2}\left(\alpha \right)\epsilon_{yy} & \text{sin}\left(\alpha \right)\cos\left(\alpha \right)\left(\epsilon_{yy}-\epsilon_{xx}\right)\\ \text{sin}\left(\alpha \right)\cos\left(\alpha \right)\left(\epsilon_{yy}-\epsilon_{xx}\right) & {\text{sin}}^{2}\left(\alpha \right)\epsilon_{xx}+\cos^{2}\left(\alpha \right)\epsilon_{yy} \end{array}\right)\text{.}$$

Let us take *α* = 30° as an example:

(8) $$\left(\frac{3}{4}\epsilon_{xx}+\frac{1}{4}\epsilon_{yy}\right)k_{x}^{2}+\left(\frac{1}{4}\epsilon_{xx}+\frac{3}{4}\epsilon_{yy}\right)k_{y}^{2}+\frac{\sqrt{3}}{2}\left(\epsilon_{yy}-\epsilon_{xx}\right)k_{x}k_{y}=\epsilon_{xx}\epsilon_{yy}k_{0}^{2}\text{.}$$

At this time, the IFCs are no longer ideal ellipses, straight lines or hyperbolae, but have the feature of distortion. The rotation of the unit cell will strongly modify the IFC shape. As a result, the propagation properties of electromagnetic waves will become more diverse by tuning the rotational angle. The twisted models can not only apply to the engineering of SPP transport but also have potential applications in the design of new active devices.

**Figure 2:**
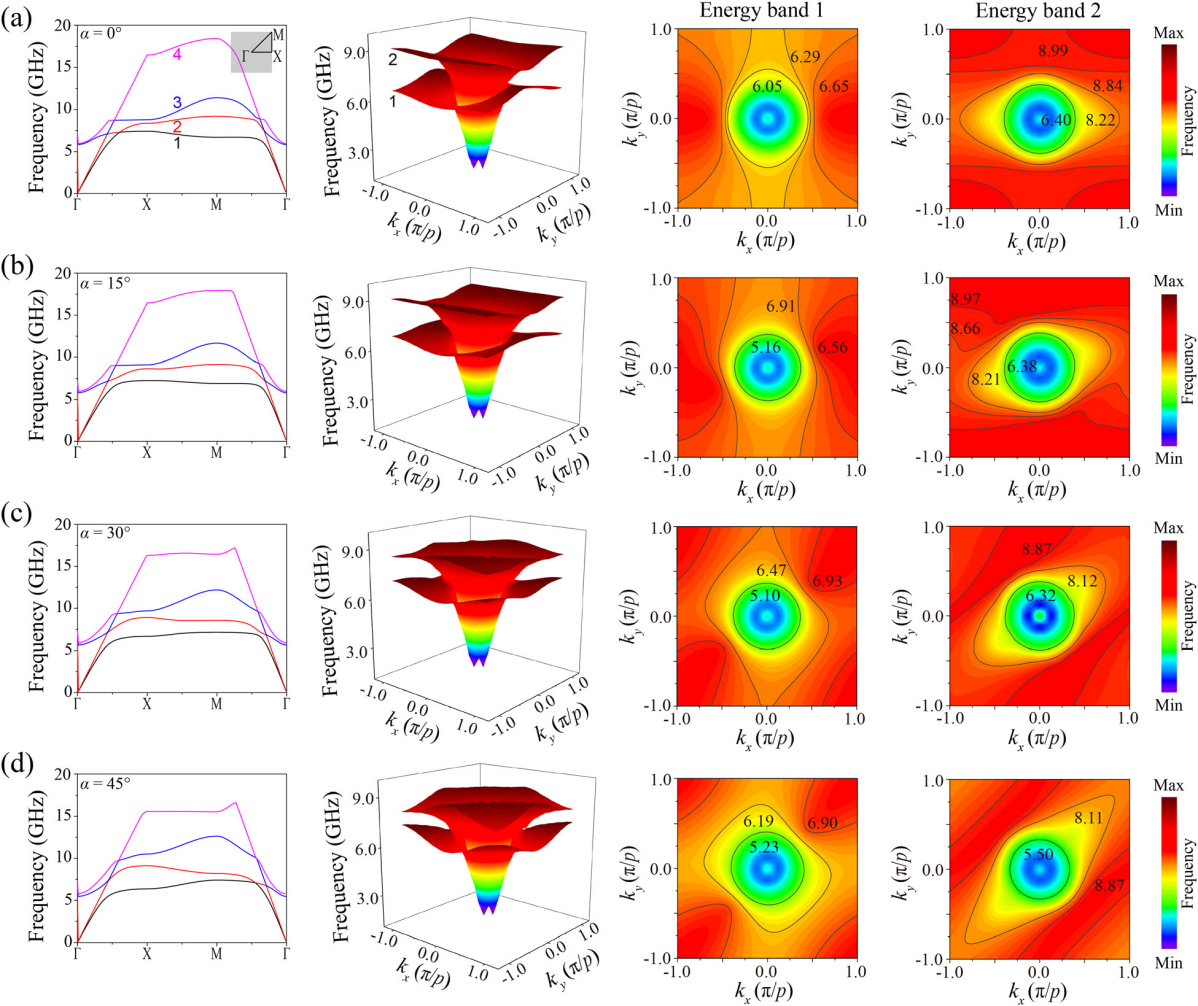
The 3D energy band diagrams/IFCs of the first two bands in the first Brillouin zone when the rotation angles are (a) 0° (the inset in the band diagram illustrates the first Brillouin zone and the highly symmetrical points), (b) 15°, (c) 30°, and (d) 45°, respectively. The unit of the frequency values in the IFCs is GHz.

To gain a better understanding of the electromagnetic characteristics of the presented HMS, we numerically calculated its band diagrams using the eigenvalue module of the commercial software Computer Simulation Technology (CST) along the highly symmetrical points in the first Brillouin zone. Based on the equation *k* = *π* × *θ*/180*p*, the values of *k*_*x*_ and *k*_*y*_ are obtained, and the 3D energy band diagrams/IFCs of the first two energy bands in the first Brillouin zone are presented in Figure 2(a)–(d) for different rotation angles, while the inset in Figure 2(a) indicates the first Brillouin zone and the highly symmetrical points. It is obvious that when the primitive cell rotates 0–45° counterclockwise, the IFCs rotate counterclockwise and the band overlap appears in the second IFC when *α* = 45°. Additionally, there are varying degrees of distortion. The presence of the extensive overlap means that there are no Lifshitz-like continuous topological transitions in the band diagrams, so the flat band is difficult to be independently excited. Fortunately, there are two ways to break the overlap to obtain a new self-collimation frequency: one is to break the extensive overlap between different energy bands, and the other is to generate a new resonance frequency excited by the incident electromagnetic waves [42]. Therefore, a new self-collimation frequency can be obtained with the appearance of new resonance modes by the counterclockwise rotation around the *z*-axis, which will simultaneously break the symmetry in the *x* and *y* directions. For example, when the unit cells rotate counterclockwise around the *z* axis, a tilted self-collimation frequency appears when *α* = 45°. Another appealing feature is that the extreme points (red areas) are no longer at the high symmetry points, which originates from the primitive cell rotation and the lattice no longer having a reflection surface, so the extreme points are not necessary at the boundary of the Brillouin zone. In order to study the underlying mechanism of these changes, we discuss the transmission spectra in the following.

**Figure 3:**
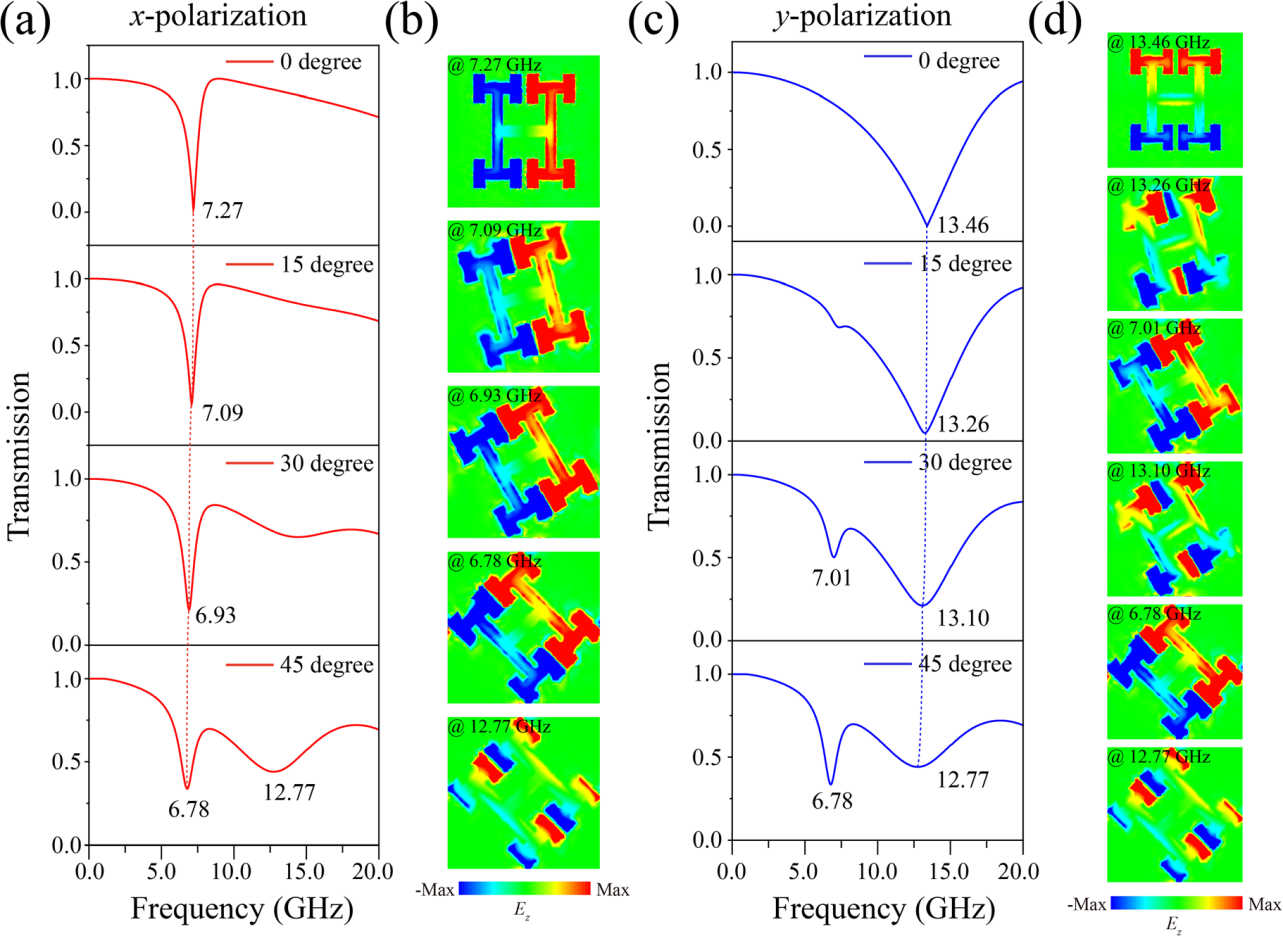
Transmittance spectra and *E_z_* field distributions. (a and c) Normalized transmittance spectra when the incident waves are polarized in the *x* and *y* directions, respectively. (b and d) *E*_*z*_ field distributions on the surface of the unit cells at the resonance valleys corresponding to (a) and (c).

As shown in Figure 3(a) and (b), a slight red shift appears in the transmission dip as the model rotates when the incident light is polarized along the *x* direction, and the coupling in the *x*-direction also rotates counterclockwise. However, a new dip appears at 12.77 GHz as an octapole when the model rotates 45°. Figure 3(c) and (d) exhibit, respectively, new resonance modes of the hexapole (13.26 GHz for *α* = 15° and 13.10 GHz for *α* = 30°) and octapole interactions along the *y* direction when the incident light is polarized in the *y*-direction. The higher-order coupling indicates that the metasurface manipulates the interaction between light and matter in new ways, and thus the suppression of radiation loss will be achieved. Besides, because the higher-order multi-dipoles and new types of interactions between the *x* and *y* directions are excited with the rotation of the unit cells, not only the energy bands rotate accordingly, but distortions of the band diagrams also appear. Therefore, the rotation operation can be used as an effective solution to obtain flat lines without distortion in the IFC, so as to achieve self-collimation frequencies.

Based on the normalized transmittance spectra discussed above, we make a quantitatively characterization of the permittivity by treating the proposed twisted HMSs as homogeneous anisotropic effective-medium slabs, and the effective permittivity can be obtained by the *S*-parameter retrieval process [62], [63], [64]. The *S* matrix can be written as: $S=\left(\begin{array}{ll} S_{11} & S_{12}\\ S_{21} & S_{22} \end{array}\right)\text{,}$ where the first and second subscripts indicate the output and input ports, respectively. Therefore, *S*_11_ and *S*_21_ denote, respectively, the reflection and transmission coefficients, which are related to the effective refractive index *n* and the impedance *z* of the dielectric slab by the following expressions:

(9) $$S_{12}^{-1}=S_{21}^{-1}=\cos\left(nkh\right)-\frac{i}{2}\left(z+\frac{1}{z}\right)\text{sin}\left(nkh\right)\text{.}$$

(10) $$S_{11}=S_{22}=\frac{i}{2}\left(\frac{1}{z}-z\right)\text{sin}\left(nkh\right)\text{.}$$

where *h* is the thickness of the dielectric slab. The effective permittivity *ε* can be calculated by ϵ=n/z as follows:

(11) $$n=\frac{1}{kh}\cos^{-1}\left[\frac{1}{2S_{21}}\left(1-S_{11}^{2}+S_{21}^{2}\right)\right],\;z=\sqrt{\frac{{\left(1+S_{11}\right)}^{2}-S_{21}^{2}}{{\left(1-S_{11}\right)}^{2}-S_{21}^{2}}}\text{.}$$

**Figure 4:**
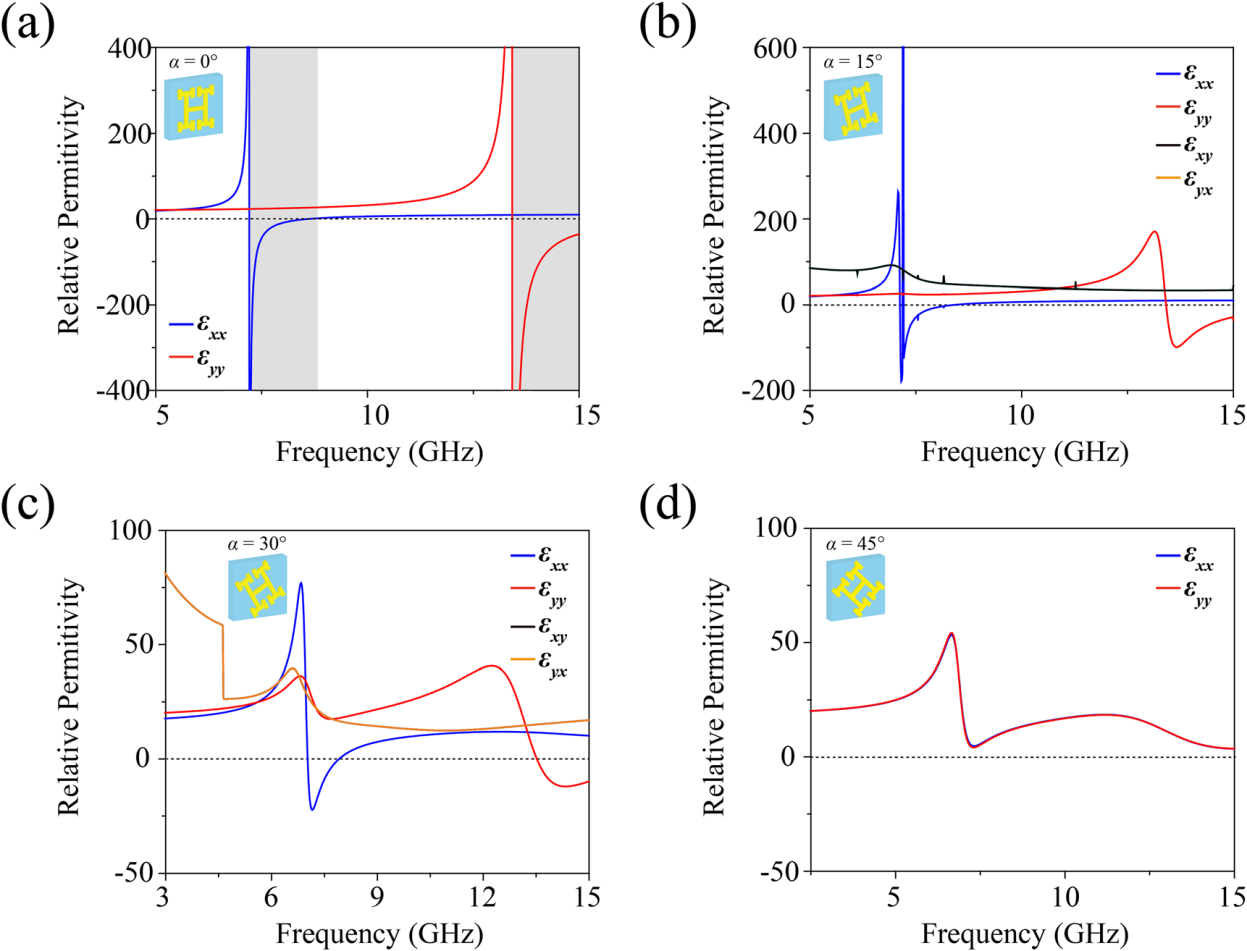
Relative permittivity obtained from EMT when the counterclockwise rotation angles of the sample are (a) *α* = 0°, (b) *α* = 15°, (c) *α* = 30°, and (d) *α* = 45°, respectively. The gray regions in (a) exhibit the hyperbolic IFCs predicted by EMT.

The corresponding calculation results are plotted in Figure 4. When the rotation angle *α* = 0° and $\epsilon_{xy}=\epsilon_{yx}=0$ , the effective permittivity only has diagonal terms, which successfully predict the effective medium properties. The gray areas (7.21–8.67 GHz and <13.42 GHz) represent the hyperbolic IFCs and topological transition frequency (7.21 GHz) where ɛ_*xx*_ × ɛ_*yy*_ < 0. However, there is obvious deviation in frequencies between the simulated IFCs and the calculated relative permittivity, mainly resulting from the constructed 2D homogeneous dielectric slab. This is due in part to the permittivity of the realistic 3D structure determined by the transcendental equation is more complicated and cannot be solved, and another cause is the small *S* parameter data in magnitude that have a great influence on the retrieval process [65]. In the case of 0° < *α* < 45° and $\epsilon_{xy}=\epsilon_{yx}\neq 0$ , the relative permittivity obtained from EMT shows good agreement with Eq. (7). It can be seen that the permittivities *ɛ*_*xx*_ and *ɛ*_*yy*_ change dramatically with the breaking of the symmetry in the *x* and *y* directions due to the rotation of the models. Besides, the off-diagonal relative permittivities *ɛ*_*xy*_ and *ɛ*_*yx*_ denote the novel interactions between the *x* and *y* directions, and thus, the energy bands twist accordingly and the distortion occurs as shown in Figure 2. Due to the symmetry of the sample, the relative permittivities *ɛ*_*xx*_ = *ɛ*_*yy*_, and *ɛ*_*xy*_ = *ɛ*_*yx*_ = 0 when *α* = 45°, which is consistent with the permittivity tensor given in Eq. (7).

### 2.3 Multi-directional SPP propagation

**Figure 5:**
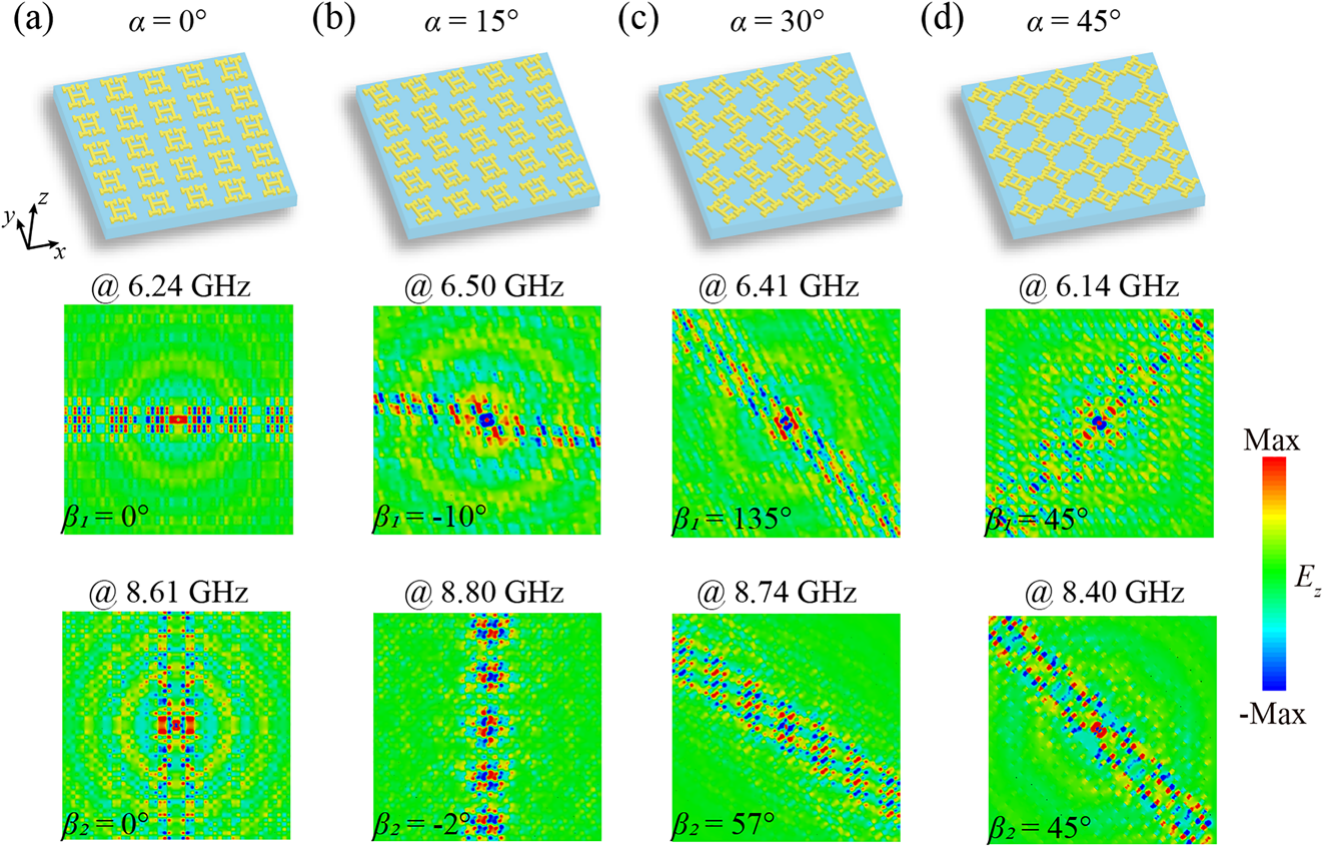
The twisted samples and *E*_*z*_ distributions at the transition frequencies when the counterclockwise rotation angles of the samples are (a) *α* = 0°, (b) *α* = 15°, (c) *α* = 30°, and (d) *α* = 45°, respectively. *β*_1_ and *β*_2_ are the counterclockwise rotation angles of the propagation directions of the SPPs with respect to the *x* and *y* axes, respectively.

As shown in the right panel in Figure 1, a microwave dipole, resonating in the *z* direction, can generate surface waves on the surface of the electromagnetic HMSs. The CST time-domain solver was used to simulate the electric field *E*_*z*_ distributions of the rotated model (*α* = 0–45°) at the respective transition frequencies of the first two energy bands as depicted in Figure 5. As the surface plasmon group velocity is perpendicular to the IFC $\left({\vec{V}}_{g}=\nabla_{\vec{k}}\omega \right)$ , self-collimation only occurs at the transition frequencies in two perpendicular directions. With the rotation of the model, the IFCs rotate counterclockwise, so the surface waves at the topological transition frequencies rotate counterclockwise accordingly. However, because of the obvious overlap and perturbation in the IFCs along with the rotation, the rotation angle of the surface waves at the topological transition frequencies is different from the twisted angle of the model when 0° < *α* < 45°. In this case, we obtain nondiffraction-like SPP transmission by searching the frequency at which the photon density of states reaches a maximum. The mismatching between the measured wave vectors and the calculated IFCs is owing to the distorted and extremely overlapped IFCs [66]. Consistent with the energy bands and IFCs described above, there are tilted lines in of the second energy band IFCs when the model rotates 45°, and therefore, there is nondiffraction transmission in the diagonal direction. Due to the high degeneracy of the self-collimating frequency, the surface waves are better confined than at other frequencies, so we choose the unit cell with *α* = 45° and *α* = 135° as the primitive cells for the following model.

### 2.4 Negative refraction

**Figure 6:**
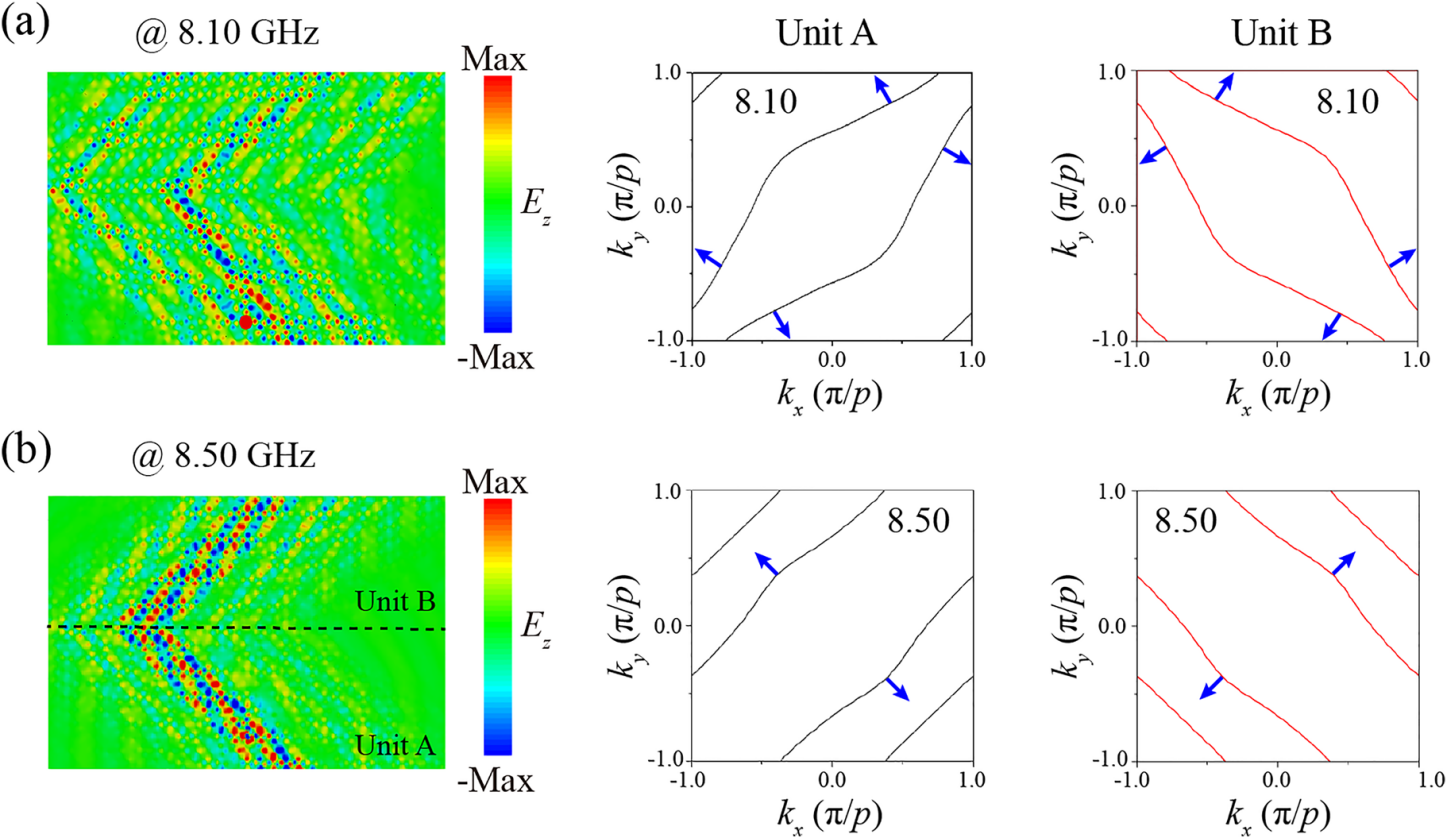
Calculated *E*_*z*_ distributions in the *xy* plane at 0.05 mm above the integrated structure and the corresponding IFCs at (a) 8.10 GHz and (b) 8.50 GHz, respectively.

Due to the rotational symmetry, compared with the model whose rotation angle is *α*, the IFC of the model with a rotation angle of *α* +90° is relatively rotated by 90°. By combining different twisted meta-resonators, SPP propagation can be redirected at the interface at will. For example, observation of negative refraction phenomenon is presented in Figure 6 when the SPPs traverse an interface between two metasurfaces whose rotation angles are 45° (unit A) and 135° (unit B), respectively. We select two typical negative refraction phenomena and the corresponding IFCs for analyzing the underlying mechanism. As illustrated in Figure 6, the bottom half of the metasurface consists of arrays of units A (29 × 8 unit cells) while the top half array is made up of units B (29 × 8 unit cells). The electric dipole is located at the center of the boundary at the bottom side.

Interestingly, at 8.10 GHz, as the unit A has a hyperbolic IFC, the excited SPPs transmit along two crossing straight lines. Therefore, for units B, the IFC rotates 90° relative to that of units A, and the surface waves rotate 90° as well. The surface waves can reach the interface along two transmission paths, and they travel in the directions perpendicular to the original trajectories after passing through the interface, forming a dual-channel negative refraction phenomenon. On the other hand, at 8.50 GHz, the IFCs of units A are four straight lines with a rotational angle of 45° with respect to the horizontal direction. Therefore, the surface waves transmit along the diagonal directions without diffraction. At this time, the photon density of states reaches a maximum, which is expressed as nondiffraction negative refraction. The proposed HMS thus provides a new method for negative refraction phenomena, anomalous wave propagation, asymmetric transmission, etc. And there will be a wider range of applications in the areas of surface optoelectronic device integration, sensors, hyperlenses, and in-plane beam steering.

## 3 Experimental results

**Figure 7:**
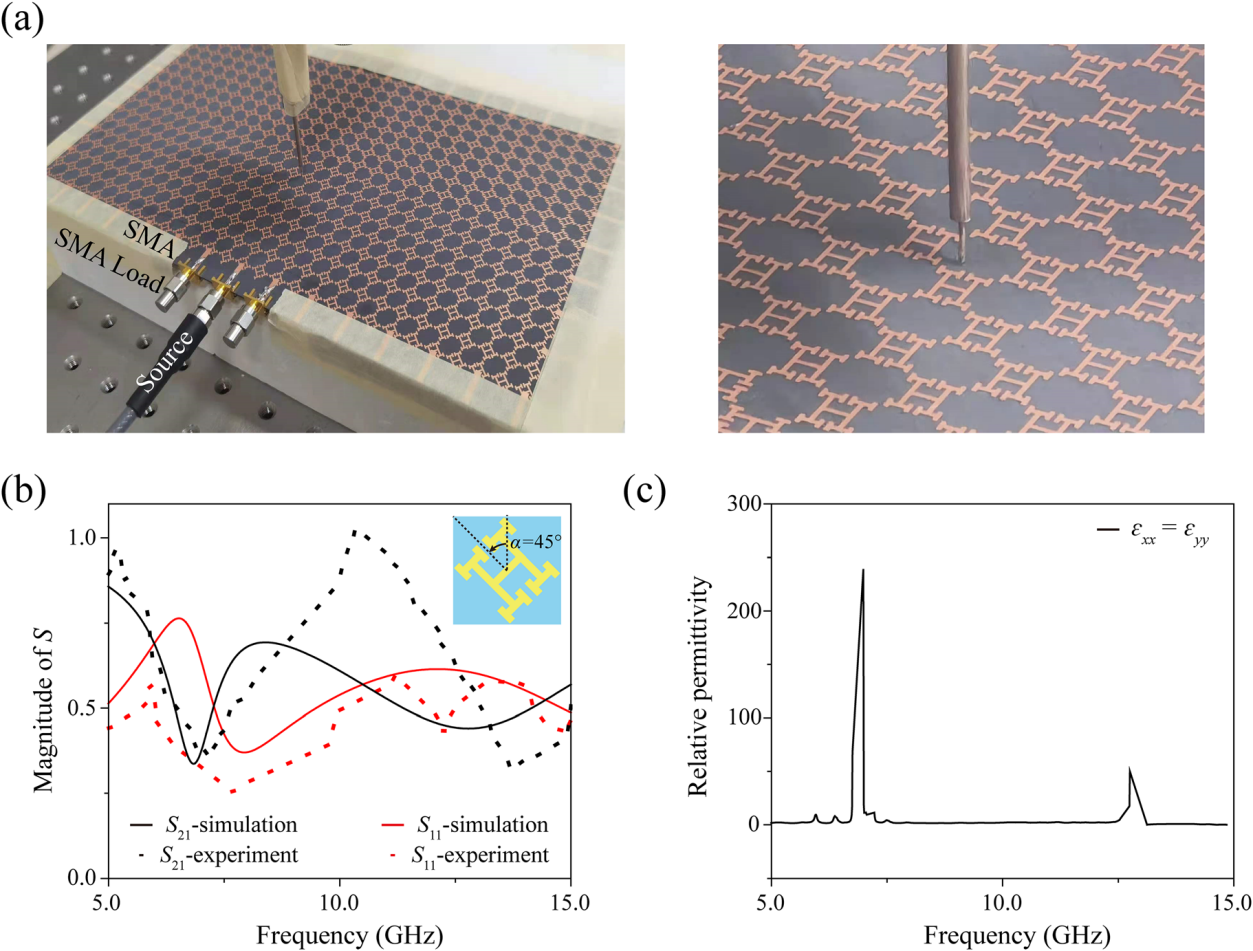
Experimental results. (a) Details of the measurement process in the microwave regime. (b) Transmission and reflection spectra of the measurements (points) and simulations (lines) when the source was polarized along the *y* axis. (c) Relative permittivity obtained from the measurements.

As shown in Figure 7, a metasurface consisting of 29 × 16 unit cells and excitation units (total size 279 × 162 mm) was fabricated. The dielectric slab was made of F_4_BM300 with a permittivity of 3.0 and *tgδ* ≤ 7 × 10^−4^ at 10 GHz. The metallic patterns were printed on the slab with 35 µm thick copper and the organic solderability preservatives antioxidation process were employed on the metal surface to prevent oxidation. The structure was designed with *p* = 9 mm, *a* = 4.5 mm, *b* = 2.6 mm, *c* = *d* = 1.6 mm, and *e* = 0.56 mm. Microstrip lines with a width of 2.4 mm and a length of 13.5 mm was designed around the structure to match the impedance of the network analyzer in order to excite surface waves. The impedance was calculated to be 50.99 Ω through ‘microstrip calculation’, which matched the impedance of the network analyzer of 50 Ω, and simulations further proved that the influence of the microstrip lines on the electric distribution could be neglected. In order to calculate and verify the effective medium property of the realistic metasurfaces, we measured the transmission and reflection data of the fabricated HMS (the counterclockwise rotation angle was *α* = 45°) as shown in Figure 7(b). Here, the experimental process led to some deviations: there was a small angle between the receiving port and the incident port when measuring the reflection spectrum, the excitation signal was scattered into air, and the noise in the experiment especially had a greater impact on the calculation process. Overall, the measured results match well with the simulation, and we can assume that the permittivity calculated from the realistic metasurface coincides with the theoretical calculations (Figure 7(c)).

**Figure 8:**
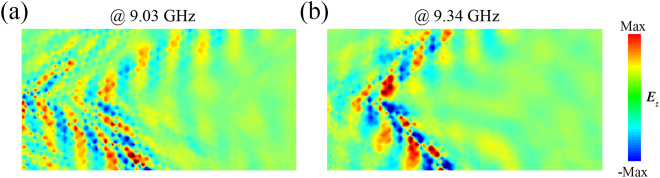
Measured *E*_*z*_ distributions in the *xy* plane at 0.05 mm above the integrated structure at (a) 9.03 GHz and (b) 9.34 GHz, respectively.

In the experiment, an SMA connector was used to produce a microwave dipole between the substrate and the metal structure to excite the SPPs, as shown in Figure 7. Since the dipole is similar to a spherical wave, in order to solve the problem of the coupling between the source and the surrounding structures, we placed two SMA loads with a nominal impedance of 50 Ω and frequency range of 0–18 GHz on both sides of the dipole. An antenna was used to detect the *z*-oriented electric field at 0.05 mm above the metasurface with a step of 2 mm along both the *x*-axis and the *y*-axis by a 3D translation platform. Compared with the *E*_*z*_ distributions in Figure 6, the measured electric fields depicted in Figure 8 agree well with our simulations. Similarly, a dual-channel phenomenon appears at 9.03 GHz. When the frequency is near 9.34 GHz, the dual-channel phenomenon disappears, and the maximum photon density of states is reached at this time, which means that nondiffracting negative refraction was realized. The experimental bandwidths of the dual-channel and nondiffraction negative refraction of the surface waves are 0.25 and 0.19 GHz, respectively. In the simulation, they are, respectively, 0.26 and 0.20 GHz, which shows excellent consistence with the experiments. However, in this situation, the results show that the corresponding frequencies between the simulations and measurements have a slight shift, which might result from the fabrication error. For example, the realistic permittivity is hard to determine, which will result in enormous loss and largely affect the working frequencies, and the thickness will also have an error of ±0.02–±0.04 mm. Overall, the measurements agree with the simulations, showing the validity of the proposed twisted HMS and the unusual negative refraction, which can be used to improve the transmission efficiency of hyperlenses and other new photonic devices.

## 4 Conclusions

We theoretically and experimentally studied a series of twisted HMSs, which were proved to deliver an efficient way to realize dual-channel and nondiffracting negative refraction. The internal mechanism of the distorted energy bands and IFCs were also analyzed. Such unique rotated HMSs will greatly increase the application range of surface waves in nondiffraction transmission, anomalous wave propagation, negative refraction, and super-resolution imaging, and offer new opportunities in developing integrated circuits and plasmonic devices.
